# Adjuvant chemotherapy benefit according to T and N stage in small bowel adenocarcinoma: a large retrospective multicenter study

**DOI:** 10.1093/jncics/pkad064

**Published:** 2023-09-29

**Authors:** Aziz Zaanan, Julie Henriques, Anthony Turpin, Sylvain Manfredi, Romain Coriat, Eric Terrebonne, Jean-Louis Legoux, Thomas Walter, Christophe Locher, Olivier Dubreuil, Simon Pernot, Chloé Vernet, Olivier Bouché, Vincent Hautefeuille, Johan Gagniere, Thierry Lecomte, David Tougeron, Thomas Grainville, Dewi Vernerey, Pauline Afchain, Thomas Aparicio

**Affiliations:** Department of Digestive Oncology, European Georges Pompidou Hospital, Assistance Publique-Hôpitaux de Paris (APHP), Université Paris Cité, Paris Cancer Institute CARPEM, Paris, France; Methodology and Quality of Life Unit in Oncology, centre hospitalier universitaire (CHU) Besançon, Hôpital Jean Minjoz, Besançon, France; Bourgogne Franche-Comté University, INSERM, Etablissement Français du Sang Bourgogne Franche-Comté, UMR1098, Interactions Hôte-Greffon-Tumeur/Ingénierie Cellulaire et Génique, Besançon, France; Department of Medical Oncology, centre hospitalier universitaire (CHU) Lille—Hôpital Claude Huriez, Lille, France; Department of Hepato-Gastroenterology and Digestive Oncology, Dijon University Hospital, EPICAD LNC UMR 1231, University of Burgundy, Dijon, France; Gastroenterology and Oncology Department, Hôpital Cochin AP-HP, Université Paris Cité, Paris, France; Gastroenterology Department, CIC 1401, centre hospitalier universitaire (CHU) Haut Lévèque, Pessac, France; Department of Hepato-Gastroenterology and Digestive Oncology, Centre Hospitalier Régional d’Orléans, Orléans, France; Department of Medical Oncology, Hospices Civils de Lyon, Lyon, France; Gastroenterology and Digestive Oncology Department, Meaux Hospital, Meaux, France; Department of Digestive Oncology, Groupe hospitalier Diaconesses Croix Saint Simon, Paris, France; Department of Medical Oncology, Institut Bergonié, Bordeaux, France; Department of Digestive Oncology, Hôpital Privé Jean Mermoz, Lyon, France; Department of Digestive Oncology and Gastroenterology, University of Reims Champagne-Ardenne (URCA), centre hospitalier universitaire (CHU) Reims, Reims, France; Department of Hepato-Gastroenterology and Digestive Oncology, Amiens University Hospital, Amiens, France; Department of Digestive and Hepatobiliary Surgery, University Hospital of Clermont-Ferrand, U1071 INSERM, Clermont-Auvergne University, Clermont-Ferrand, France; Department of Hepato-Gastroenterology and Digestive Oncology, University Hospital of Tours, Tours University, U1069 INSERM “Nutrition, Croissance et Cancer”, Tours, France; Department of Hepato-Gastroenterology, centre hospitalier universitaire (CHU) de Poitiers, Poitiers, France; Department of Gastroenterology, Pontchaillou Hospital, Rennes 1 University; INSERM U1242, Rennes, France; Methodology and Quality of Life Unit in Oncology, centre hospitalier universitaire (CHU) Besançon, Hôpital Jean Minjoz, Besançon, France; Bourgogne Franche-Comté University, INSERM, Etablissement Français du Sang Bourgogne Franche-Comté, UMR1098, Interactions Hôte-Greffon-Tumeur/Ingénierie Cellulaire et Génique, Besançon, France; Department of Oncology, Saint Antoine Hospital, Paris, France; Gastroenterology and Digestive Oncology Department, Saint Louis Hospital, AP-HP, Université Paris Cité, Paris, France

## Abstract

**Background:**

Small bowel adenocarcinoma is a rare cancer, and the role of adjuvant chemotherapy for localized disease is still debated.

**Methods:**

This retrospective multicenter study included all consecutive patients who underwent curative surgical resection for localized small bowel adenocarcinoma between 1996 and 2019 from 3 French cohort studies. Prognostic and predictive factors of adjuvant chemotherapy efficacy were analyzed for disease-free survival and overall survival. The inverse probability of treatment weighting method was applied in the Cox regression model using the propensity score derived from multivariable logistic regression.

**Results:**

A total of 354 patients were included: median age, 63.5 years; duodenum location, 53.5%; and tumor stage I, II, and III in 31 (8.7%), 144 (40.7%), and 179 (50.6%) patients, respectively. The adjuvant chemotherapy was administered in 0 (0%), 66 (48.5%), and 143 (80.3%) patients with stage I, II, and III, respectively (*P* < .0001). In the subgroup analysis by inverse probability of treatment weighting method, a statistically significant disease-free survival and overall survival benefit in favor of adjuvant chemotherapy was observed in high-risk stage II (T4 and/or <8 lymph nodes examined) and III (T4 and/or N2) but not for low-risk stage II (T3 and ≥8 lymph nodes examined) and III (T1-3/N1) tumors (*P*_interaction_ < .05). Furthermore, tumor location in jejunum and ileum was also a statistically significant predictive factor of response to adjuvant chemotherapy in stage II and III tumors (*P*_interaction_ < .05).

**Conclusion:**

In localized small bowel adenocarcinoma, adjuvant chemotherapy seems to provide a statistically significant survival benefit for high-risk stage II and III tumors and for jejunum and ileum tumor locations.

Small bowel cancers are rare diseases, accounting for approximately 5% of all gastrointestinal cancers, with the predominance of small bowel adenocarcinoma that has increased in incidence across recent years (1). The incidence of small bowel adenocarcinoma varies by geographic area, with rates that appear to be higher in North America and Western Europe and lower in Asian countries (1). Exploratory methods have been improved for these difficult-to-diagnose tumors, but patients often have nonspecific symptoms, which can therefore delay diagnosis (2). Most of these primary tumors arise in the duodenum (55%-60%), followed by jejunum (25%-30%) and ileum (15%) locations, and the median age at diagnosis is approximately 60 years, with a male predominance (2). Predisposing diseases are found in approximately 20%-30% including familial adenomatous polyposis, Lynch syndrome, Peutz–Jeghers syndrome, Crohn disease, and celiac disease (3-5). Studies based on prospective cohorts have shown that approximately 40% of patients have localized disease at diagnosis, and their prognosis is worse on average than for other related malignancies, including colon cancer at the same tumor stage (4-6). In this context, adjuvant chemotherapy could be interesting to eradicate the residual microscopic disease responsible for disease recurrence and thus improve patient survival. According to the American National Cancer Database, the use of adjuvant chemotherapy has increased from 24.2% in 1998 to 43.4% in 2011 (7), and fluoropyrimidine with or without oxaliplatin are the most common regimens based on efficacy of these drugs in advanced small bowel adenocarcinoma and by analogy to the adjuvant treatment of colon cancer (8).

However, given the rarity of small bowel adenocarcinoma and the lack of randomized studies, there is no clear evidence of efficacy regarding the adjuvant treatment after small bowel adenocarcinoma resection (4). Data from the literature are conflicting presumably because of a lack of patient stratification for confounding factors and specification of treatment modalities in most studies (4). The first data from retrospective studies have found no benefit in adjuvant chemotherapy after curative surgical resections (9-15). More recently, another study based on the American National Cancer Database revealed a decrease in the risk of death in favor to adjuvant chemotherapy for patients with stage III small bowel adenocarcinoma and a trend of improvement in overall survival with adjuvant chemotherapy for those with stage II T4 tumors (7). These data highlight the need to identify tumor factors of high-risk of recurrence to guide adjuvant treatment as it is recommended for colon cancer. Therefore, most recommendations for treatment of patients with localized small bowel adenocarcinoma come from expert agreements or from analogies to the management of colon cancer patients. The French guidelines updated in 2022 recommend, with a low level of evidence, adjuvant chemotherapy for stage III and stage II with T4 tumor (16). For the National Comprehensive Cancer Network guidelines updated in 2020, adjuvant chemotherapy with fluoropyrimidine and oxaliplatin is recommended for stage III, while for stage II, adjuvant chemotherapy with fluoropyrimidine with or without oxaliplatin is recommended for those with high-risk features of recurrence such as T4 and/or less than 8 lymph nodes examined (17). Furthermore, in stage III colon cancer, duration and regimen of adjuvant chemotherapy are being guided by stratification of patients into low (T1-3 and N1) and high (T4 and/or N2) risk groups based on the IDEA (International Duration Evaluation of Adjuvant Chemotherapy) study (18). However, this tumor stratification of low- and high-risk T and N stage groups for adjuvant chemotherapy in localized small bowel adenocarcinoma has never been evaluated.

In this study, we aim to evaluate the benefit of adjuvant chemotherapy in small bowel adenocarcinoma in terms of disease-free survival (DFS) and overall survival according to tumor stage and high-risk features defined by T4 and/or less than 8 lymph nodes examined for stage II and by T4 and/or N2 for stage III.

## Methods

### Study participants

This retrospective multicenter study included all consecutive patients with histologically confirmed and resectable small bowel adenocarcinoma treated between 1996 and 2019 with surgery alone or followed by adjuvant chemotherapy based on fluoropyrimidine with or without oxaliplatin planned for 6 months in French centers. For each patient, adjuvant treatment and the regimen of chemotherapy were decided in a multidisciplinary tumor board. The study population included patients from 3 cohorts: AGEO-PHRC cohort between 1996 and 2008 studied by Zaanan et al. and Aparicio et al. (19,20), ARCAD-NADEGE cohort between 2009 and 2012 studied by Aparicio et al. (5), and AGEO-COLOGREL cohort between 2013 and 2019 not published yet. Patient files were retrieved from tumor registries of pathology departments and information system medical programs in each center, using the International Statistical Classification of Diseases and Related Health Problems version 10 (ICD-10) international codes C17.0 (duodenum), C17.1 (jejunum), and C17.2 (ileum). Exclusion criteria were individuals aged younger than 18 years, residual tumor R1 or R2 status for the surgical resection of localized small bowel adenocarcinoma, tumor stage 0 (carcinoma in situ), adjuvant treatment based on drugs other than fluoropyrimidine with or without oxaliplatin or radiotherapy, and death within 30 days after surgery. This study was conducted in accordance with the Declaration of Helsinki. The AGEO-PHRC and ARCAD-NADEGE cohort studies were previously authorized by the ethics committee Ile de France II No. ID-RCB: 2008-A01058-47 (20,5), and the AGEO-COLOGREL cohort was more recently approved (DR-2020-260 n° 920274).

### Treatment and outcome

Data were collected on relevant demographic data, tumor characteristics including the number of lymph nodes examined and vascular emboli, lymphatic invasion, and perinervous invasion (21) for stage II; predisposing disease or known genetic syndrome; adjuvant chemotherapy regimens; local and distant disease recurrences; and death. Routine follow-up was similar to colon cancer consisting of physical examination, biological tests, and computed tomography scan (or ultrasonography) every 3-6 months for at least 5 years. The data were updated in June 2021.

### Statistical analysis

Baseline clinical and pathological variables were described in overall population, according to stage and adjuvant chemotherapy with median and range for continuous variables and frequencies with percentages for qualitative variables. Differences in baseline characteristics according to postoperative management were assessed using Wilcoxon test for continuous variables and the χ^2^ test or Fisher exact test for categorical variables.

The primary endpoint was the association between adjuvant chemotherapy and survival (DFS and overall survival) in stage II and III small bowel adenocarcinoma separately. DFS was defined as the time elapsed from diagnosis to the first recurrence or death from any cause. Patients alive without relapse were censored at the date of last follow-up. Overall survival was defined as the time elapsed from diagnosis to death from any cause. Patients alive were censored at the date of last follow-up. Survivals and follow-up were estimated by Kaplan–Meier and reverse Kaplan–Meier methods, respectively, and described with median and 95% confidence interval (CI). Log-rank tests were used to compare survivals curves.

Association between baseline characteristics and survivals was estimated with univariable Cox proportional hazards regression models, and the hazard ratio (HR) with 95% confidence interval was provided. Variables with a *P* value of .10 or less in univariate analysis were eligible for the Cox multivariable regression model, which was constructed according to the Peduzzi rule of 1 independent variable entered for 10 events. Correlation between variables was assessed, and as variable of interest, adjuvant chemotherapy was forced in the multivariable model.

A propensity score method was used to limit potential bias because of confounding parameters unbalanced between patients untreated or treated with adjuvant chemotherapy in stage II and III separately. Univariable logistic regression was first used to model the probability of having adjuvant chemotherapy, and then variables with a *P* value less than .15 were introduced into multivariable model after correlation checking. The area under the curve (AUC) and Hosmer–Lemeshow test statistic were estimated. The inverse probability of treatment weighting method was applied in the Cox regression model using the propensity score derived from multivariable logistic regression to assess the association between adjuvant chemotherapy and survivals.

As a secondary endpoint, the association between adjuvant chemotherapy and survival was analyzed in subgroups according to relevant characteristics and risk groups. The forest plots with *P*_interaction_ value and hazard ratio with 90% confidence interval obtained with inverse probability of treatment weighting method applied in the Cox regression model were provided. In this study, high-risk features were defined by T4 and/or less than 8 lymph nodes examined for stage II small bowel adenocarcinoma as recommended by National Comprehensive Cancer Network guidelines (17) and by T4 and/or N2 (≥3 positive lymph nodes) for stage III small bowel adenocarcinoma as it is dichotomized in stage III colon cancer by IDEA consortium (18). Conversely, low-risk tumors were defined by T3 and at least 8 lymph nodes examined for stage II and by T1-3 and N1 (<3 positive lymph nodes) for stage III small bowel adenocarcinoma.

A *P* value of less than .05 was considered statistically significant. All statistical tests were 2-sided, and *P* values were not adjusted for multiple testing because of the exploratory context of the study. All analyses were performed using SAS software version 9.3 (SAS Institute, Cary, NC, USA) and R software version 4.1.

## Results

### Study population

**Figure 1.**
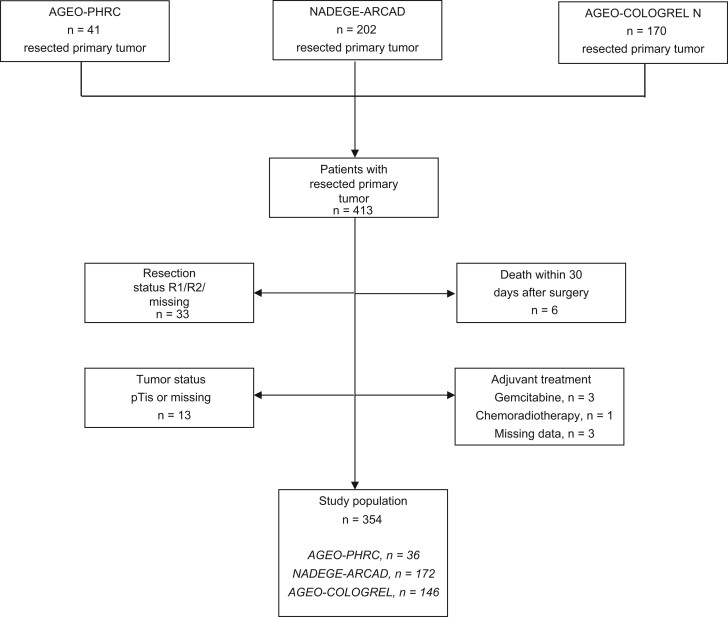
Flow chart.

Among the 413 patients who underwent a surgical resection for localized small bowel adenocarcinoma from AGEO-PHRC (n = 41), ARCAD-NADEGE (n = 202), and AGEO-COLOGREL (n = 170) cohorts, 59 were excluded because of tumor stage 0 (Tis), incomplete tumor resection (R1 or R2), adjuvant treatment based on gemcitabine chemotherapy or radiotherapy, death within 30 postoperative days, or missing data regarding the tumor resection or adjuvant treatment (Figure 1, Flow Chart). Demographic and clinical characteristics of the study population (n = 354) are listed in Table 1 and stratified by the tumor stage I (n = 31), stage II (n = 144), and stage III (n = 179). Duodenal location and predisposing disease were more frequent in earlier tumor stage. The administration of adjuvant chemotherapy was more frequent in stage III than in stage II tumor, while patients with stage I tumor were exclusively treated with surgery alone (Table 1). As expected, after a median follow-up of 5.2 years (95% CI = 4.7 to 6.0 years), early tumor stage was associated with a longer DFS and overall survival: the 5-year DFS rates were 78%, 61%, and 42%, and the 5-year overall survival rates were 88%, 76%, and 58%, for tumor stage I, II, and III, respectively (Supplementary Figure 1, available online).

**Table 1.** Characteristics of the study population^a^

Characteristics	Overall population	Stage I	Stage II	Stage III	*P*
	(n = 354)	(n = 31)	(n = 144)	(n = 179)	
Sex, No. (%)					.06
Male	197 (55.65)	21 (67.74)	70 (48.61)	106 (59.22)	
Female	157 (44.35)	10 (32.26)	74 (51.39)	73 (40.78)	
Age at diagnosis, No. (%)					
Younger than 75 y	294 (83.05)	25 (80.65)	123 (85.42)	146 (81.56)	.61
Median age (range), y	63.49 (20.32-89.12)	61.33 (41.16-86.97)	63.72 (20.32-87.55)	63 (24.25-89.12)	.76
Primary tumor location, No. (%)					.006
Duodenum	189 (53.54)	24 (77.42)	62 (43.36)	103 (57.54)	
Jejunum	98 (27.76)	4 (12.90)	50 (34.97)	44 (24.58)	
Ileum	66 (18.70)	3 (9.68)	31 (21.68)	32 (17.88)	
Missing	1	0	1	0	
Predisposing disease, No. (%)					.046
No	244 (72.84)	18 (64.29)	90 (67.16)	136 (78.61)	
Yes	91 (27.16)	10 (35.71)	44 (32.84)	37 (21.39)	
Crohn disease	39 (11.64)	3 (10.71)	18 (13.43)	18 (10.40)	
Lynch syndrome	42 (12.54)	5 (17.86)	19 (14.18)	18 (10.40)	
Familial adenomatous polyposis, No. (%)	3 (0.90)	1 (3.57)	2 (1.49)	0 (0)	
Coeliac disease	7 (2.09)	1 (3.57)	5 (3.73)	1 (0.58)	
Missing	19	3	10	6	
Adjuvant chemotherapy, No. (%)					<.0001
No	136 (39.42)	31 (100.00)	70 (51.47)	35 (19.66)	
Yes	209 (60.58)	0 (0)	66 (48.53)	143 (80.34)	
Missing	9	0	8	1	
Adjuvant chemotherapy regimen, No. (%)					.02
FOLFOX or CAPOX	179 (85.65)	0 (0)	53 (80.30)	126 (88.11)	
LV5FU2 or CAPECITABINE	30 (14.35)	0 (0)	13 (19.70)	17 (11.89)	

a CAPOX = capecitabine plus oxaliplatin; FOLFOX = 5-fluorouracil and oxaliplatin.

### Survival according to adjuvant chemotherapy in stage II

In stage II small bowel adenocarcinoma, 48.5% (66 of 136) of patients were treated with adjuvant chemotherapy including the FOLFOX (5-fluorouracil and oxaliplatin) or CAPOX (capecitabine and oxaliplatin) regimens in 80.3% and fluoropyrimidine alone (5-fluorouracil or capecitabine) in 19.7% (Table 1).

The characteristics of patients with stage II small bowel adenocarcinoma according to the administration of adjuvant chemotherapy are shown in Table 2. Patients treated with adjuvant chemotherapy were statistically significantly more likely to be younger and to have high-risk (T4 and/or <8 lymph nodes examined) and vascular emboli, lymphatic invasion, and perinervous invasion–positive tumors (Table 2). In univariate and multivariate analysis, adjuvant chemotherapy was not statistically significantly associated with an improvement in DFS or overall survival, while the high-risk subgroup was associated with a statistically significant worse overall survival and a trend for worse DFS as compared with the low-risk subgroup (Table 3).

**Table 2.** Characteristics of patients with stage II small bowel adenocarcinoma with and without adjuvant chemotherapy

Characteristics	Stage II population	No adjuvant chemotherapy	With adjuvant chemotherapy	*P*
	(n = 136)	(n = 70)	(n = 66)	
Sex, No. (%)				.87
Male	65 (47.79)	33 (47.14)	32 (48.48)	
Female	71 (52.21)	37 (52.86)	34 (51.52)	
Age at diagnosis, No. (%)				
Younger than 75 y	116 (85.29)	55 (78.57)	61 (92.42)	.02
Median age (minimum-maximum), y	63.79 (20.32-87.55)	67.28 (29.28-87.55)	62.03 (20.32-78.93)	.01
Primary tumor location, No. (%)				.35
Duodenum	61 (45.19)	35 (50.00)	26 (40.00)	
Jejunum	46 (34.07)	20 (28.57)	26 (40.00)	
Ileum	28 (20.74)	15 (21.43)	13 (20.00)	
Missing	1	0	1	
Predisposing disease, No. (%)				.75
No	85 (66.93)	44 (65.67)	41 (68.33)	
Yes	42 (33.07)	23 (34.33)	19 (31.67)	
Crohn disease	16 (12.60)	5 (7.46)	11 (18.33)	
Lynch syndrome	19 (14.96)	14 (20.90)	5 (8.33)	
Familial adenomatous polyposis	2 (1.57)	0 (0)	2 (3.33)	
Coeliac disease	5 (3.94)	4 (5.97)	1 (1.67)	
Missing	9	3	6	
Differentiation, No. (%)				
Well and moderate	105 (88.24)	54 (88.52)	51 (87.93)	.92
Low	14 (11.76)	7 (11.48)	7 (12.07)	
Missing	17	9	8	
Perforation, No. (%)				1.00
No	102 (95.33)	55 (94.83)	47 (95.92)	
Yes	5 (4.67)	3 (5.17)	2 (4.08)	
Occlusion, No. (%)				.31
No	81 (75.00)	42 (71.19)	39 (79.59)	
Yes	27 (25.00)	17 (28.81)	10 (20.41)	
Vascular emboli, lymphatic invasion, and perinervous invasion, No. (%)				.02
No	59 (62.11)	37 (72.55)	22 (50)	
Yes	36 (37.89)	14 (27.45)	22 (50)	
pT, No. (%)				.002
pT3	90 (66.18)	55 (78.57)	35 (53.03)	
pT4	46 (33.82	15 (21.43	31 (46.97	
Lymph nodes examined, No. (%)				.03
<8	44 (36.67)	18 (27.69)	26 (47.27)	
≥8	76 (63.33)	47 (72.31)	29 (52.73)	
Missing	1	1	0	
Risk group, No. (%)				<.0001
Low, T3 and ≥8 lymph nodes examined	49 (38.89)	38 (56.72)	11 (18.64)	
High, T4 and/or <8 lymph nodes examined	77 (61.11)	29 (43.28)	48 (81.36)	
Missing	10	3	7	

**Table 3.** Univariate and multivariate Cox models for disease-free survival and overall survival in the stage II population

		Disease-free survival								Overall survival							
		Univariate				Multivariate				Univariate				Multivariate			
		No. (events)	HR	95% CI	*P* ^a^	No. (events)	HR	95% CI	*P* ^a^	No. (events)	HR	95% CI	P^a^	No. (events)	HR	95% CI	*P* ^a^
Sex	Male	65 (24)	1		.46					65 (17)	1		.39				
	Female	71 (24)	0.81	0.46 to 1.43						71 (15)	0.74	0.37 to 1.48					
Age, y	Younger than 75	116 (38)	1		.07	107 (37)	1		0.27	116 (25)	1		.02	107 (25)	1		.14
	75 and older	20 (10)	1.91	0.95 to 3.85		19 (9)	1.53	0.72 to 3.27		20 (7)	2.76	1.16 to 6.58		19 (7)	2.01	0.79 to 5.13	
Tumor location	Duodenum	61 (20)	1		.90					61 (12)	1		.88				
	Jejunum	46 (19)	1.04	0,56 to 1.96						46 (13)	1.15	0.52 to 2.54					
	Ileum	28 (8)	0.86	0.38 to 1.96						28 (7)	1.25	0.49 to 3.19					
Predisposing disease	No disease	85 (35)	1		.14					85 (21)	1		.74				
	Disease	42 (12)	0.61	0.32 to 1.18						42 (11)	1.13	0.55 to 2.35					
pT	T3	90 (28)	1		.09^b^					90 (18)	1		.04^a^				
	T4	46 (20)	1.66	0.93 to 2.95						46 (14)	2.08	1.02 to 4.25					
Lymph nodes examined	<8	44 (20)	1		.07^b^					44 (16)	1		.09^c^				
	≥8	76 (22)	0.57	0.31 to 1.04						76 (15)	0.54	0.27 to 1.10					
Risk group	T3 and N ≥ 8	49 (13)	1		.04	49 (13)	1		0.09	49 (7)	1		.02	49 (7)	1		.005
	T4 or N < 8	77 (33)	1.98	1.04 to 3.76		77 (33)	2.26	1.14 to 4.48		77 (25)	2.75	1.19 to 6.38		77 (25)	3.82	1.48 to 9.83	
Differentiation	Well and moderately	105 (35)	1		.25					105 (24)	1		.046^d^				
	Low	14 (6)	1.66	0.70 to 3.95						14 (6)	2.51	1.02 to 6.19					
Vascular emboli, lymphatic invasion, and perinervous invasion	No	59 (15)	1		.44					59 (8)	1		.06^c^				
	Yes	36 (12)	1.35	0.63 to 2.89						36 (11)	2.4	0.96 to 6.00					
Occlusion	No	81 (21)	1		.06^c^					81 (16)	1		.30				
	Yes	27 (12)	2.00	0.98 to 4.07						27 (8)	1.57	0.67 to 3.68					
Perforation	No	102 (31)	1		.72					102 (23)	1		.79				
	Yes	5 (1)	0.7	0.10 to 5.13						5 (1)	1.31	0.17 to 9.76					
Adjuvant chemotherapy	No	70 (28)	1		.49	67 (27)	1		.21	70 (19)	1		.69	67 (19)	1		.23
	Yes	66 (20)	0.82	0.46 to 1.45		59 (19)	0.67	0.35 to 1.26		66 (13)	0.86	0.43 to 1.76		59 (13)	0.62	0.28 to 1.37	

a Variables with a *P* value of .10 or less in univariate analysis were eligible for the Cox multivariable regression model. CI = confidence interval; DFS = disease-free survival; HR = hazard ratio.

b pT and lymph nodes examined were not included in the multivariate model because these variables are used to construct the high- and low-risk groups (nonindependent factors).

c Multivariate analysis was not performed for these variables because of a relatively high rate of missing data (DFS, 21% for occlusion data; overall survival, 30% for vascular emboli, lymphatic invasion, and perinervous invasion data).

d This variable (differentiation) was not retained for the multivariate model because 3 variables were already selected (age, risk group, and adjuvant chemotherapy) of the 32 events observed (Peduzzi rule of 1 independent variable for 10 events). As variable of interest, adjuvant chemotherapy was forced in the multivariable model.

The propensity score was built with all relevant variables unbalanced between patients with and without adjuvant chemotherapy. The multivariable logistic regression including age at diagnosis and risk group to estimate the probability to receive adjuvant chemotherapy exhibited an AUC equal to 0.76, which means that the model succeeds to predict patients receiving adjuvant chemotherapy in 76% of cases (Supplementary Table 1, available online).

**Figure 2.**
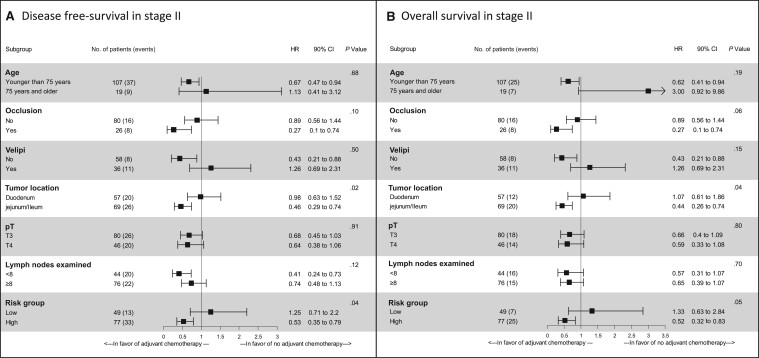
Forest plot for disease-free survival **(A)** and overall survival **(B)** in stage II population by the inverse probability of treatment weighting method. High-risk group: pT4 or less than 8 lymph nodes examined; low-risk group: pT3 and 8 or more lymph nodes examined; lower confidence interval and upper confidence interval: 90%. The inverse probability of treatment weighting method was applied in the Cox regression model using the propensity score derived from multivariable logistic regression to assess the association between adjuvant chemotherapy and survival. Two variables were not tested in this model because of the very unbalanced distribution: perforation (yes vs no: 5 vs 102) and differentiation (low vs well or moderate: 14 vs 102). CI = confidence interval; HR = hazard ratio; VELIPI = vascular emboli, lymphatic invasion, and perinervous invasion.

In inverse probability of treatment weighting method analysis, adjuvant chemotherapy was associated with a statistically significant improvement of DFS (HR = 0.67, 90% CI = 0.46 to 0.99; *P* = .04) and a trend for an improvement of overall survival (HR = 0.65, 90% CI = 0.41 to 1.02; *P* = .06). In subgroup analysis, a statistically significant differential effect on DFS was observed in the low- and high-risk groups (*P*_interaction_  = .04), with a higher benefit from adjuvant chemotherapy observed in high-risk tumor (HR = 0.53, 90% CI = 0.35 to 0.79) as compared with low-risk tumor (HR = 1.25, 90% CI = 0.71 to 2.20) (Figure 2). The same result was observed for overall survival (*P*_interaction_ = .05) (Figure 2). Interestingly, the tumor location was also a predictive marker of response to adjuvant chemotherapy with a statistically significant *P*_interaction_ value (.02 for DFS and .04 for overall survival). Patients with jejunum and ileum tumor had a higher DFS (HR = 0.46, 90% CI = 0.29 to 0.74) and overall survival (HR = 0.44, 90% CI = 0.26 to 0.74) benefit in favor of adjuvant chemotherapy as compared with patients with duodenum location (DFS: HR = 0.98, 90% CI = 0.63 to 1.52; overall survival: HR = 1.07, 90% CI = 0.61 to 1.86) (Figure 2).

### Survival according to adjuvant chemotherapy in stage III

In stage III small bowel adenocarcinoma, 80.3% (143 of 178) of patients were treated with adjuvant chemotherapy including the FOLFOX or CAPOX regimens in 88.1% and fluoropyrimidine alone (5-fluorouracil or capecitabine) in 11.9% (Table 1).

The characteristics of patients with stage III small bowel adenocarcinoma according to the administration of adjuvant chemotherapy are listed in Table 4. There was no statistically significant difference apart from age with younger patients in adjuvant chemotherapy group. In univariate and multivariate analysis, adjuvant chemotherapy was not associated with a statistically significant improvement in either DFS or overall survival, while high-risk subgroup (T4 and/or N2) was associated with a statistically significant worse DFS and overall survival as compared with low-risk subgroup (Table 5).

**Table 4.** Characteristics of patients with stage III small bowel adenocarcinoma with and without adjuvant chemotherapy

Characteristics	Stage III population	No adjuvant chemotherapy	With adjuvant chemotherapy	*P*
	(n = 178)	(n = 35)	(n = 143)	
Sex, No. (%)				.48
Male	106 (59.55)	19 (54.29)	87 (60.84)	
Female	72 (40.45)	16 (45.71)	56 (39.16)	
Age at diagnosis, No. (%)				
Younger than 75 y	145 (81.46)	18 (51.43)	127 (88.81)	<.0001
Median age (range), y	63 (24.25-89.12)	72.42 (24.25-89.12)	62.69 (30.89-88.50)	
Primary tumor location, No. (%)				.35
Duodenum	103 (57.87)	24 (68.57)	79 (55.24)	
Jejunum	43 (24.16)	6 (17.14)	37 (25.87)	
Ileum	32 (17.98)	5 (14.29)	27 (18.88)	
Predisposing disease, No. (%)				.43
No	135 (78.49)	25 (73.53)	110 (79.71)	
Yes	37 (21.51)	9 (26.47)	28 (20.29)	
Crohn disease	18 (10.47)	3 (8.82)	15 (10.87)	
Lynch syndrome	18 (10.47)	6 (17.65)	12 (8.70)	
Coeliac disease	1 (0.58)	0 (0)	1 (0.72)	
Missing	6	1	5	
pT, No. (%)				.44
T1-T2	12 (3.39)	4 (11.43)	8 (5.63)	
T3	80 (45.20)	14 (40.00)	66 (46.48)	
T4	85 (48.02)	17 (48.57)	68 (47.89)	
Missing	1	0	1	
pN, No. (%)				.14
1	95 (57.23)	22 (68.75)	73 (54.48)	
2	71 (42.77)	10 (31.25)	61 (45.52)	
Missing	12	3	9	
Risk group, No. (%)				.85
T1-3 and N1	55 (31.98)	11 (33.33)	44 (31.65)	
T4 and/or N2	117 (68.02)	22 (66.67)	95 (68.35)	
Missing	6	2	4	

**Table 5.** Univariate and multivariate Cox models for disease-free survival and overall survival in the stage III population^a^

		Disease-free survival								Overall survival							
		Univariate				Multivariate^b^^,^^c^				Univariate				Multivariate			
		No. (events)	HR	95% CI	*P*	No. (events)	HR	95% CI	*P*	No. (events)	HR	95% CI	*P*	No. (events)	HR	95% CI	*P*
Sex	Male	106 (57)	1		.89					106 (39)	1		.77				
	Female	72 (39)	0.97	0.65 to 1.46						72 (31)	1.07	0.67 to 1.72					
Age, y	Younger than 75	145 (76)	1		.10					145 (53)	1		<.01	142 (53)	1		.06
	75 and older	33 (20)	1.51	0.92 to 2.49						33 (17)	2.24	1.29 to 3.90		30 (15)	1.82	0.97 to 3.44	
Tumor location	Duodenum	103 (52)	1		.33					103 (38)	1		.30				
	Jejunum	43 (22)	0.99	0.60 to 1.64						43 (15)	0.86	0.47 to 1.56					
	Ileum	32 (22)	1.43	0.87 to 2.36						32 (17)	1.44	0.81 to 2.55					
Predisposing disease	No	135 (74)	1		.33					135 (53)	1		.39				
	Yes	37 (20)	0.78	0.48 to 1.28						37 (15)	0.78	0.44 to 1.38					
pT^b^	T1-T3	92 (42)	1		.001					92 (31)	1		.02				
	T4	85 (54)	1.97	1.31 to 2.95						85 (39)	1.76	1.09 to 2.82					
pN	N1	95 (38)	1		<.01					95 (25)	1		<.01				
	N2	71 (51)	2.35	1.54 to 3.58						71 (39)	2.40	1.45 to 3.96					
Risk group	T1-3 and N1	55 (19)	1		<.01	55 (19)	1		<.01	55 (44)	1		<.01	55 (43)	1		<.01
	T4 and/or N2	117 (75)	2.74	1.65 to 4.54		117 (75)	2.76	1.66 to 4.57		117 (95)	2.60	1.42 to 4.77		117 (55)	2.56	1.40 to 4.70	
Adjuvant chemotherapy	No	35 (20)	1		.30	33 (18)	1		.37	35 (18)	1		.06	33 (16)	1		.46
	Yes	143 (76)	0.77	0.47 to 1.26		139 (76)	0.79	0.47 to 1.32		143 (52)	0.59	0.35 to 1.02		139 (52)	0.79	0.43 to 1.47	

a Variables with a *P* value of .10 or less in univariate analysis were eligible for the Cox multivariable regression model. CI = confidence interval; DFS = disease-free survival; HR = hazard ratio.

b pT and lymph nodes examined were not included in the multivariate model because these variables are used to construct the high- and low-risk groups (nonindependent factors).

c As variable of interest, adjuvant chemotherapy was forced in the multivariable model.

The propensity score was built with all relevant variables unbalanced between patients with and without adjuvant chemotherapy. The multivariable logistic regression including age at diagnosis and pN stage to estimate the probability to receive adjuvant chemotherapy exhibited an AUC equal to 0.67, which means that the model succeeds to predict patients receiving adjuvant chemotherapy in 67% of cases (Supplementary Table 2, available online).

**Figure 3.**
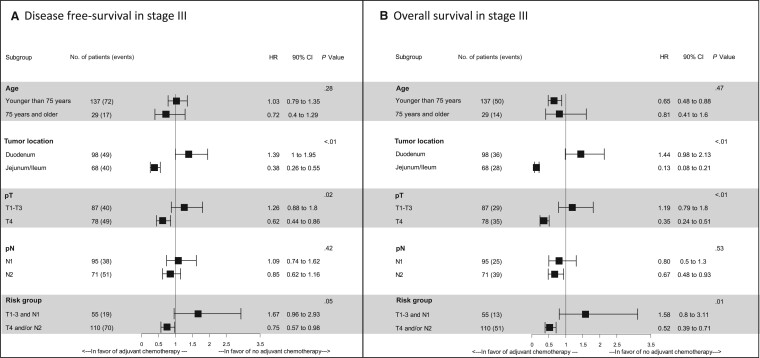
Forest plot for disease-free survival **(A)** and overall survival **(B)** in stage III population by the inverse probability of treatment weighting method. High-risk group: pT4 and/or N2; low-risk group: pT1-3/N1; lower confidence interval and upper confidence interval: 90%. The inverse probability of treatment weighting method was applied in the Cox regression model using the propensity score derived from multivariable logistic regression to assess the association between adjuvant chemotherapy and survival. CI = confidence interval; HR = hazard ratio.

In inverse probability of treatment weighting method analysis, adjuvant chemotherapy was associated with a statistically significant improvement for overall survival (HR = 0.68, 90% CI = 0.49 to 0.94; *P* = .02) but not for DFS (HR = 0.93, 90% CI = 0.70 to 1.24; *P* = .61). In subgroup analysis, the benefit of adjuvant chemotherapy on DFS was greater in the high-risk tumors (HR = 0.75, 90% CI = 0.57 to 0.98) and pT4 (HR = 0.62, 90% CI = 0.44 to 0.86) as compared with low-risk tumors (HR = 1.67, 90% CI = 0.96 to 2.93) and pT1-T3 (HR = 1.26, 90% CI = 0.88 to 1.80) with a statistically significant *P*_interaction_ value (.05 for risk group and .02 for pT stage) (Figure 3). The same result was observed for overall survival with a statistically significant *P*_interaction_ value (.01 for risk group and <.01 for pT group) (Figure 3). As observed for stage II small bowel adenocarcinoma, tumor location was also a predictive marker of response to adjuvant chemotherapy in stage III small bowel adenocarcinoma with a statistically significant *P*_interaction_ value (<.01 for DFS and overall survival). Patients with jejunum and ileum tumor had a higher DFS (HR = 0.38, 90% CI = 0.26 to 0.55) and overall survival (HR = 0.13, 90% CI = 0.08 to 0.21) benefit in favor to adjuvant chemotherapy as compared with patients with duodenum location (DFS: HR = 1.39, 90% CI = 1.00 to 1.95; overall survival: HR = 1.44, 90% CI = 0.98 to 2.13) (Figure 3).

## Discussion

To our knowledge, this is the first published report evaluating in a large population the benefit of adjuvant chemotherapy of patients with localized small bowel adenocarcinoma by identification of low- and high-risk T and N stage groups, in terms of DFS and overall survival. The question remains a subject of debate because of the lack of clear demonstration of the benefit of adjuvant treatment for this disease (4). Our large retrospective study extending over a period of more than 20 years showed that adjuvant chemotherapy was proposed for a majority of patients with a stage III tumor (80.3%) and only for approximately half (48.5%) of those with a stage II tumor. Interestingly, for stage III patients, those who did not receive adjuvant chemotherapy were older, whereas for stage II tumors, those who received chemotherapy not only were statistically significantly younger but also had a more frequently high-risk tumor. Analysis of the clinical and pathological features in stage II and III small bowel adenocarcinoma indicated that T and N stage were the most important contributors to DFS and overall survival benefit from adjuvant chemotherapy. This unbalanced distribution of poor prognostic characteristics in the adjuvant chemotherapy group may explain the lack of survival benefit in favor to adjuvant chemotherapy in univariate analysis, thus underlining the interest of a propensity score to limit these biases.

For stage II tumor, the subgroup analysis by inverse probability of treatment weighting method model showed that patients with high-risk tumors (pT4 or <8 lymph nodes examined) seemed to benefit from adjuvant chemotherapy, whereas the survival gain for low-risk patients was less or even nonexistent (statistically significant *P*_interaction_ tests). These results must be put into perspective with previous data from retrospective or meta-analysis and database studies that have not shown a survival benefit for patients treated with adjuvant chemotherapy, probably due to the lack of stratification on the clinicopathologic factors (4). In the study of Ecker et al. (7) based in the American National Cancer Database revealed a trend of improvement in overall survival with adjuvant chemotherapy for those with stage II T4 tumors. In our study, pT4 or less than 8 lymph nodes examined  taken into account separately did not allow precise identification of the subgroups of patients most sensitive to adjuvant chemotherapy. The combination of these 2 factors, which is observed in 61.1% of stage II small bowel adenocarcinoma, seems to better select patients for adjuvant chemotherapy. Furthermore, patients aged younger than 75 years or with a tumor diagnosed in occlusion or negative for vascular emboli, lymphatic invasion, and perinervous invasion criteria seemed also to benefit from adjuvant chemotherapy, but the *P*_interaction_ test was not statistically significant.

For stage III tumor, the subgroup analysis by inverse probability of treatment weighting method showed that high-risk tumors (pT4 and/or N2), as well as pT4 tumors considered separately, seemed to benefit from adjuvant chemotherapy (statistically significant *P*_interaction_ tests), whereas the survival gain for patients at low-risk (pT1-3/N1) or with a pT1-T3 tumor was less or even nonexistent. These data may explain the contradictory results of previous studies that did not consider these pathological characteristics to assess the value of adjuvant chemotherapy for stage III tumors. As for colon cancer, stratification on pT and pN to distinguish low and high risk for stage III small bowel adenocarcinoma could help guide the choice of adjuvant treatment.

Interestingly, this study also showed that tumor location was a predictive factor of response to adjuvant chemotherapy for stage II and III small bowel adenocarcinoma. Indeed, patients with tumors of the jejunum or ileum benefited from adjuvant chemotherapy based on fluoropyrimidine alone or with oxaliplatin in terms of DFS and overall survival, whereas tumors of the duodenum did not seem to respond to this treatment. In accordance with these results, 2 recent meta-analysis of large studies focusing on resectable duodenal small bowel adenocarcinoma of any stage failed to show any survival benefit of adjuvant chemotherapy (9,10). One hypothesis would be that duodenal small bowel adenocarcinomas have more phenotypic and molecular characteristics of pancreatic-biliary or gastric adenocarcinoma, while jejunal and ileal small bowel adenocarcinomas are more molecularly similar to colon cancer (22) and therefore may cause greater sensitivity to fluoropyrimidine with or without oxaliplatin.

Our results should be interpreted with caution owing to the retrospective nature of the study and the heterogeneity of our real-life population treated in different centers and over a long period of time. Findings for statistically significant survival benefit for high-risk stage II and III tumors were not observed in the population not adjusted for prognostic factors. However, analyses were then based on individual data considering precise clinical and pathological characteristics from this large series of patients. This approach allowed us the production of a propensity score to limit bias because of nonrandomized data and unbalanced characteristics between patients with and without adjuvant chemotherapy. These results need to be confirmed as the conclusions reached from this study are based on data modeling with the aim to eliminate any bias due to unbalanced confounding factors between the groups. Furthermore, translational analyses are planned for these retrospective cohorts to assess the prognostic and predictive value of molecular markers in the response to adjuvant chemotherapy. In this context, microsatellite instability, which is a molecular phenotype related to a deficient DNA mismatch repair system, may play a major role to guide adjuvant chemotherapy; In localized colon cancer, DNA mismatch repair system and/or microsatellite instability may predict resistance to 5-fluorouracil alone, while the addition of oxaliplatin would restore the efficacy of adjuvant chemotherapy (23).

In conclusion, adjuvant chemotherapy in resectable small bowel adenocarcinoma remains debated in the absence of randomized trials. Our study was able to highlight that adjuvant chemotherapy provided a statistically significant survival benefit in patients with high-risk stage II (T4 and/or <8 lymph nodes examined) and III (T4 and/or N2) tumors. The primary tumor location was also identified as a predictive factor of response to adjuvant chemotherapy by showing a statistically significant gain in survival restricted to patients with jejunum and ileum tumors. These results deserve to be confirmed by a randomized trial such as the BALLAD trial (NCT02502370) that is currently ongoing.

## Supplementary Material

pkad064_Supplementary_Data

## Data Availability

The data that support the findings of this study are available from the corresponding author upon request.
