# Supplementary material for: Adjuvant chemotherapy benefit according to T and N stage in small bowel adenocarcinoma: a large retrospective multicenter study
Source: JNCI Cancer Spectr. 2023 Sep 29;7(5):pkad064. doi: 10.1093/jncics/pkad064 (PMC10582691; doi:10.1093/jncics/pkad064)

Supplementary Table 1.

Propensity score to estimate the probability to receive adjuvant chemotherapy in stage II small bowel adenocarcinoma

		N	N of patients with adjuvant CT	Univariate logistic regression			Multivariate logistic regression		
				OR	95%CI	P value	OR	95%CI	P value
**Gender**	male	65	32	1		0.88			
	female	71	34	0.95	0.48-1.86				
**Age at diagnosis**	continuous	136	66	0.97	0.94-0.99	0.01	0.96	0.93-0.99	<0.01
**Tumor location**	Duodenum	61	26	1		0.36			
	ileum	28	13	1.17	0.48-2.87				
	Jejunum	46	26	1.75	0.81-3.79				
**Predisposing disease**	No	85	41	1		0.75			
	yes	42	19	0.89	0.42-1.87				
**pT**	T3	90	35	1		0.002*			
	T4	46	31	3.25	1.54-6.86				
**Differenciation**	well/moderately	105	51	1		0.92			
	low	14	7	1.06	0.35-3.23				
**Perforation**	No	102	47	1		0.79			
	Yes	5	2	0.78	0.13-4.87				
**Occlusion**	No	81	39	1		0.32			
	Yes	27	10	0.63	0.26-1.55				
**VELIPI**	No	59	22	1		0.03☩			
	Yes	36	22	2.64	1.13-6.20				
**LNE**	<8	44	26	1		0.03*			
	>=8	76	29	0.43	0.20-0.92				
**Risk group**	low	49	11	1		<0.01	1		<0.01
	high	77	48	5.72	2.53-12.91		6.54	2.77-15.46	

Abbreviation: LNE, lymph nodes examined; VELIPI (vascular emboli, lymphatic invasion and perinervous invasion); CT, chemotherapy, SBA, small bowel adenocarcinoma; OR, Odds ratio; CI, Confidence interval

A propensity score method was used to limit potential bias due to confounding parameters unbalanced between patients untreated or treated with adjuvant chemotherapy. Univariable logistic regression was first used to model the probability of having adjuvant chemotherapy and then variables with p value < 0.15 were introduced into multivariable model after correlation checking.

* pT and LNE were not included in the multivariate model because these variables are used to construct the high and low risk groups (non independant factors)

☩ VELIPI was not included in the multivariate model because of high rate of missing data (30%)

The multivariable logistic regression including age at diagnosis and risk group to estimate the probability to receive adjuvant chemotherapy exhibited an area under the curve (AUC) equal to 0.76

Supplementary Table 2.

Propensity score to estimate the probability to receive adjuvant chemotherapy in stage III small bowel adenocarcinoma

		N	N of patients with adjuvant CT	Univariate logistic regression			Multivariate logistic regression		
				OR	95%CI	P value	OR	95%CI	P value
**Gender**	Male	106	87	1		0.48			
	Female	72	56	0.76	0.36-1.61				
**Age at diagnosis**	continuous	178	143	0.95	0.92-0.98	<0.01	0.96	0.92-0.99	0.01
**Tumor location**	Duodenum	103	79	1		0.36			
	Ileum	32	27	1.64	0.57-4.73				
	Jejunum	43	37	1.87	0.71-4.97				
**Predisposing disease**	No	135	110	1		0.43			
	Yes	37	28	0.71	0.30-1.68				
**pT**	T1-T3	92	74	1		0.94			
	T4	85	68	0.97	0.46-2.04				
**pN**	N1	95	73	1		0.15	1		0.29
	N2	71	61	1.84	0.81-4.18		1.57	0.68-3.66	
**Risk group**	T1-3 and N1	55	44	1		0.85			
	T4 and/or N2	117	95	1.08	0.48-2.42				

Abbreviation: CT, chemotherapy, SBA, small bowel adenocarcinoma; OR, Odds ratio; CI, Confidence interval

A propensity score method was used to limit potential bias due to confounding parameters unbalanced between patients untreated or treated with adjuvant chemotherapy. Univariable logistic regression was first used to model the probability of having adjuvant chemotherapy and then variables with p value < 0.15 were introduced into multivariable model after correlation checking.

The multivariable logistic regression including age at diagnosis and pN stage to estimate the probability to receive adjuvant chemotherapy exhibited an area under the curve (AUC) equal to 0.67

Supplementary Figure 1.

Disease-free survival (A) and overall survival (B) of patients with small bowel adenocarcinoma according to tumor stage I, II and III

A B

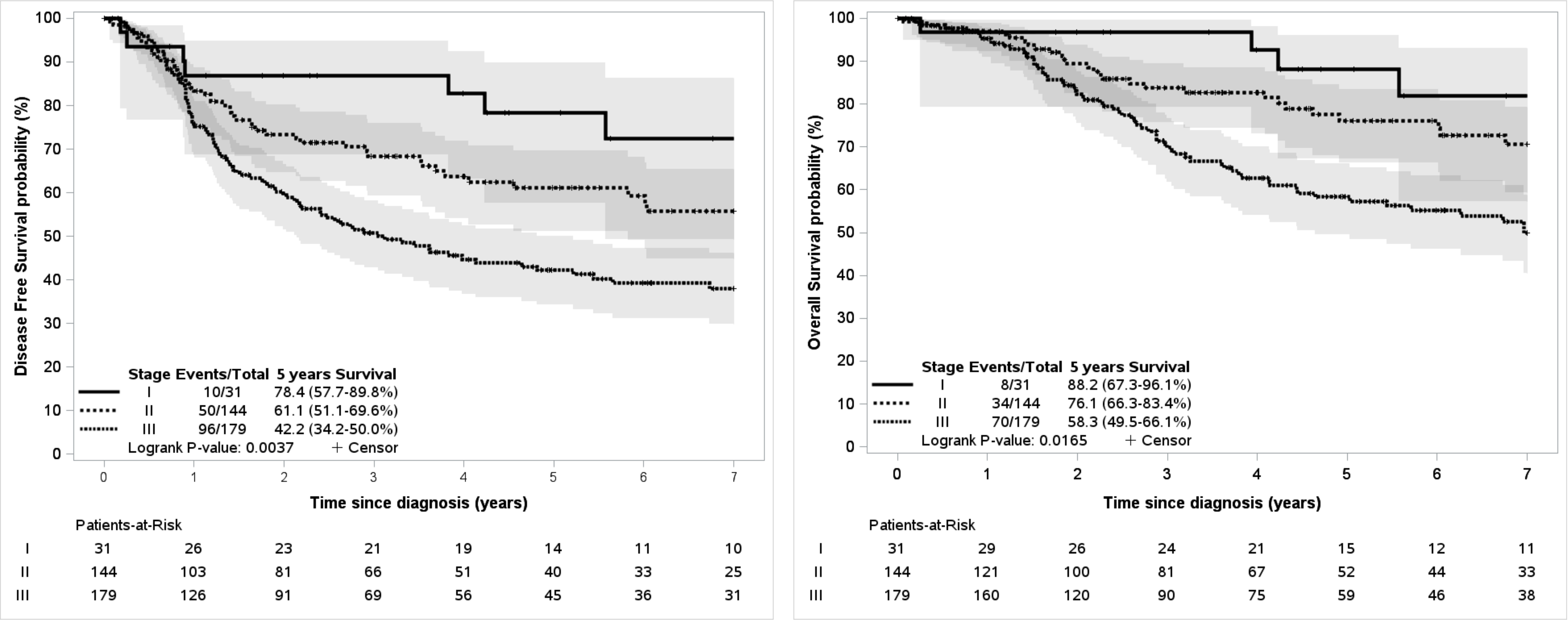

Supplement: pkad064_Supplementary_Data [file pkad064_supplementary_data.pdf]
